# Smooth Muscle-Like Tissue Constructs with Circumferentially Oriented Cells Formed by the Cell Fiber Technology

**DOI:** 10.1371/journal.pone.0119010

**Published:** 2015-03-03

**Authors:** Amy Y. Hsiao, Teru Okitsu, Hiroaki Onoe, Mahiro Kiyosawa, Hiroki Teramae, Shintaroh Iwanaga, Tomohiko Kazama, Taro Matsumoto, Shoji Takeuchi

**Affiliations:** 1 Institute of Industrial Science, The University of Tokyo, Tokyo, Japan; 2 ERATO Takeuchi Biohybrid Innovation Project, Japan Science and Technology Agency, Tokyo, Japan; 3 Faculty of Teacher Education, Shumei University, Chiba, Japan; 4 Department of Functional Morphology, Division of Cell Regeneration and Transplantation, Nihon University School of Medicine, Tokyo, Japan; Texas A&M University, UNITED STATES

## Abstract

The proper functioning of many organs and tissues containing smooth muscles greatly depends on the intricate organization of the smooth muscle cells oriented in appropriate directions. Consequently controlling the cellular orientation in three-dimensional (3D) cellular constructs is an important issue in engineering tissues of smooth muscles. However, the ability to precisely control the cellular orientation at the microscale cannot be achieved by various commonly used 3D tissue engineering building blocks such as spheroids. This paper presents the formation of coiled spring-shaped 3D cellular constructs containing circumferentially oriented smooth muscle-like cells differentiated from dedifferentiated fat (DFAT) cells. By using the cell fiber technology, DFAT cells suspended in a mixture of extracellular proteins possessing an optimized stiffness were encapsulated in the core region of alginate shell microfibers and uniformly aligned to the longitudinal direction. Upon differentiation induction to the smooth muscle lineage, DFAT cell fibers self-assembled to coiled spring structures where the cells became circumferentially oriented. By changing the initial core-shell microfiber diameter, we demonstrated that the spring pitch and diameter could be controlled. 21 days after differentiation induction, the cell fibers contained high percentages of ASMA-positive and calponin-positive cells. Our technology to create these smooth muscle-like spring constructs enabled precise control of cellular alignment and orientation in 3D. These constructs can further serve as tissue engineering building blocks for larger organs and cellular implants used in clinical treatments.

## Introduction

Smooth muscle cells are indispensable parts of many organs of the gastrointestinal, cardiovascular, urinary, and reproductive systems [1,2]. They are often spatially organized in both longitudinal and circumferential architectures around the outer layers of these visceral organs, and they are mainly responsible for the involuntary contraction (constriction/closing) and relaxation (dilation/opening) of these organs [2–4]. In particular, circumferentially organized smooth muscle cells contribute to the proper functioning of many organs. For example, it is necessary for smooth muscle cells to be oriented circumferentially to generate radially symmetrical contractions and relaxations in the gastrointestinal tract to produce a peristaltic wave that forces food through the tract [4]. Circumferentially oriented smooth muscle cells around blood vessels also contract or relax to control the diameter and in turn regulate blood flow and blood pressure [2,4–7]. Therefore, 3D cell culture and control of cellular orientation are of particular importance in engineering tissues containing smooth muscles. With the recent advancements in microscale technologies, spherical cellular aggregates such as spheroids are easy to form and have been widely used as building blocks for more complex macroscopic tissue assembly [8–11]. Although the tissue-like spheroids can theoretically be assembled into any arbitrary structure [8,9], the microscopic cellular orientation in the spherical building blocks cannot be precisely controlled.

**Fig 1:**
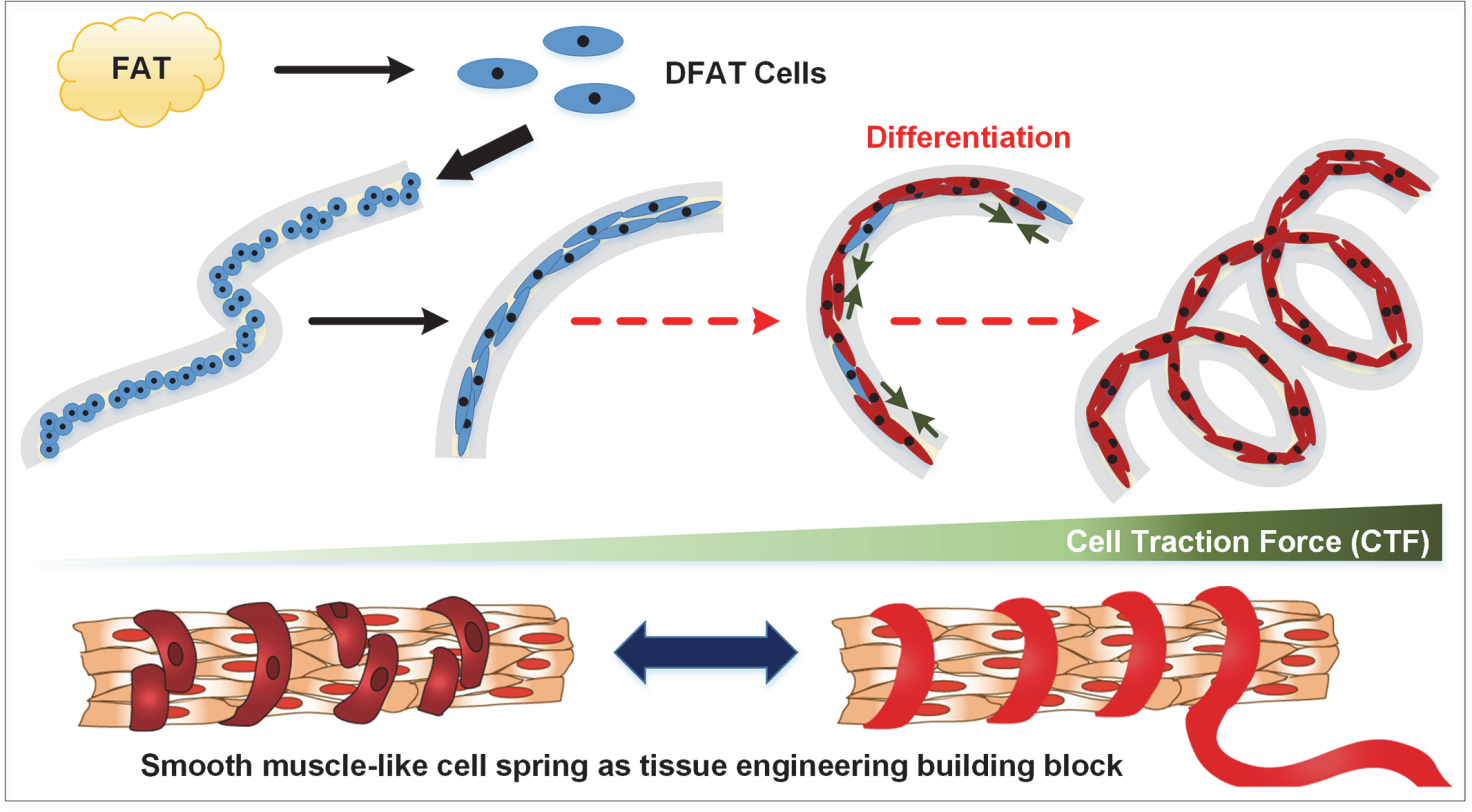
Overall concept of the study. DFAT cells derived from adipocytes are encapsulated into the center core of alginate fibers and allowed to form into cell fiber. DFAT cell fiber is subsequently induced for differentiation into smooth muscle cell lineage in induction media containing TGF-β. As cells’ inherent cell traction force increases during this process, linear cell fiber self-assembles to coiled spring structure. Such smooth muscle-like cell springs may serve as tissue engineering building blocks.

In this study, we control the orientation of cells and form circumferentially oriented smooth muscle-like tissue constructs at the micrometer scale. We utilize the cell fiber technology previously reported by our group [12]; this technology encapsulates adherent cells into the core region of a hydrogel core-shell microfiber, allowing the cells to grow, migrate, make cell-cell contact, and form into fiber-shaped tissue construct called “cell fiber.” We first encapsulate multipotent de-differentiated fat (DFAT) cells into the center core region of alginate microfibers (Fig. 1). Cells naturally elongate to the axial direction inside the high aspect ratio microenvironment of the long microfibers fabricated by a microfluidic device. DFAT cells derived from adipocytes isolated from adipose tissue through a dedifferentiation ceiling culture process can be expanded in abundance with high purity without gene manipulations, and thus provides greater efficacy and safety in clinical applications [13,14]. After the cells aligned to form into fiber structures, they are subsequently induced to differentiate into the smooth muscle cell lineage. During this differentiation process as cells’ inherent traction forces [15,16] increase, DFAT cell fibers self-assemble into smooth muscle cell-like uniformly coiled spring structures with circumferentially oriented cells that serve as useful models for circumferential smooth muscles. This simple method takes advantage of cells’ natural tendency to align and self-assemble into micro-scale 3D spring constructs without laborious pre-patterning of proteins or subsequent assembling manipulations that may bring undesired chemical and mechanical stresses to the cells. Here, we investigate the requirements for extracellular matrix (ECM) proteins to maintain fiber structure, characterize various parameters of the spring structures, and finally demonstrate expression of smooth muscle specific markers in the fibers.

## Materials and Methods

### DFAT Cell Preparation and Culture

All animal experiments were performed according to the United States National Institute of Health Guide for the Care and Use of Laboratory Animals (NIH Publication No. 85–23, revised 1996) with approval by the Animal Experiment Committee of Nihon University School of Medicine. DFAT cells were prepared using mature adipocytes isolated from the inguinal fat pads of male Japanese White rabbits (age > 6 months; body weight: 2.0–2.5 kg, CLEA Japan, Tokyo, Japan) according to the method described previously [17]. Briefly, approximately 1 gm of adipose tissue was minced and digested with 0.1% (w/v) collagenase type I (Koken, Tokyo, Japan) for 1 hour at 37°C with gentle agitation. The top floating layer containing unilocular adipocytes was collected after filtration and centrifugation at 135 g for 3 minutes. The isolated adipocytes were placed in T12.5 cell culture flasks (NUNC, Roskilde, Denmark) completely filled with Dulbecco’s Modified Eagle Medium (DMEM) (Life Technologies, Carlsbad, CA, USA) supplemented with 20% FBS (JRH Bioscience, Lenexa, KS, USA) with 5 × 10^4^ cells per flask and cultured in a humidified incubator with 5% CO_2_ at 37°C. The adipocytes floated and adhered to the top ceiling surface of the flask during the following days, and on day 7 the flask was inverted with the old media replaced with normal amount of fresh media. Media was changed every 3 days until these DFAT cells reached confluence. Multipotency of these DFAT cells were confirmed by observing that they differentiated into lineage of adipocyte, osteocyte, chondrocyte, or smooth muscle cell under the established condition for each differentiation induction [13,14].

DFAT cells were routinely cultured in general growth media composed of DMEM (Sigma-Aldrich Japan, Tokyo, Japan) supplemented with 10% (v/v) heat-inactivated fetal bovine serum (HI-FBS, Sigma-Aldrich Japan, Tokyo, Japan) and 1% (v/v) penicillin-streptomycin (P/S, Sigma-Aldrich Japan, Tokyo, Japan). When the cells were roughly 70% confluent, they were split at 1:3 subculture ratio. Cells were used for experiments before they reached passage 10. All cells were maintained in a humidified incubator with 5% CO_2_ at 37°C.

### Device Fabrication

A microfluidic double co-axial device is composed of two pulled glass capillary tubes assembled in tandem and joined by custom made three-way connectors as previously described [12]. A glass capillary tube (outer diameter = 1 mm, inner diameter = 0.6 mm) was pulled to generate two sections with 300 μm sharp tips. These then served as the two inner co-axial microchannels inside rectangular glass tubes (outer diameter = 1.4 mm, inner diameter = 1 mm) that were responsible for guiding the outer streams. All components were assembled and joined by custom made connectors fabricated by stereolithography. The inlets and outlets were finally connected to syringes through Teflon tubes. The device was thoroughly rinsed with 70% ethanol followed by 145 mM sodium chloride (NaCl, Wako Pure Chemical Industries, Osaka, Japan) solution before use.

### Hydrogel Core-Shell Microfiber Fabrication

To encapsulate DFAT cells suspended in ECM proteins in the core region of alginate fibers, three solutions were required: 1) core stream: a solution of DFAT cells suspended in ECM proteins at 1 × 10^7^ cells/ml, 2) shell stream: a solution of 1.5 wt% sodium alginate (80∼120 cP, Wako Pure Chemical Industries, Osaka, Japan), and 3) sheath stream: a solution of 100 mM calcium chloride (CaCl_2_, Kanto Chemicals, Tokyo, Japan) with 3 wt% sucrose (Nacalai Tesque, Kyoto, Japan). Seven different compositions of ECM proteins were used for the core stream solution: 1) *PCol*: 2 mg/ml pepsin-solubilized bovine type I collagen, neutralized in DMEM low glucose (AteloCell^TM^, DME-02, Atelocollagen, KOKEN, Japan), 2) *ACol-H*: 5 mg/ml acid-solubilized bovine type I collagen (AteloCell^TM^, IAC-50, Native collagen, KOKEN, Japan), neutralized in Hanks’ buffered solution containing NaHCO_3_ and HEPES to a final concentration of 4 mg/ml, 3) *ACol-L*: ACol-H solution diluted with PBS (-) at 1:1 volume ratio to achieve a final concentration of 2 mg/ml, 4) *Fib-L*: fibrinogen derived from bovine plasma (F8630-1G, Sigma-Aldrich Japan, Tokyo, Japan) dissolved at 33.3 mg/ml in 20 mM HEPES buffered saline, and adjusted to 5.5 mg/ml by diluting with a solution consisting of 10 mM HEPES + 100 mM sodium gluconate + 45 mM NaCl, 5) *Fib-M*: same as Fib-L, but with the final fibrinogen concentration adjusted to 11.0 mg/ml, 6) *Fib-H*: same as Fib-L, but with the final fibrinogen concentration adjusted to 16.5 mg/ml, and 7) *ACol-Fib*: 1:1 mixture of ACol-H solution containing 100 mM sodium gluconate and 33.3 mg/ml fibrinogen (F8630-1G, Sigma-Aldrich Japan, Tokyo, Japan) solution. Two different compositions of 1.5 wt% sodium alginate were used for the shell stream solution: 1) *Alg*: 1.5 wt% sodium alginate in 145 mM NaCl solution (used when the core stream solution does not contain fibrinogen), and 2) *Alg-Fib*: 1:1 mixture of 3 wt% sodium alginate and Fib-L solution (used when the core stream solution contains fibrin). Unless otherwise specified, all fibers used in the experiments were fabricated using the ACol-Fib core stream solution and the Alg-Fib shell stream solution.

The shell stream solution was first introduced into the device by a syringe pump at 150 μl/min and immediately followed by the sheath stream at 2.4 ml/min by a second syringe pump. Once fabrication of the fiber consisting of calcium alginate alone was confirmed to be stable, the cells suspended in ECM proteins were similarly introduced into the device as the core stream at 50 μl/min using a third syringe pump for a total of 40 seconds. By introducing all three streams into the device simultaneously, approximately 3.33 × 10^5^ DFAT cells were encapsulated inside calcium alginate shell fibers. The fabricated fibers (each around 0.5 meter in length) were temporarily collected in a conical tube filled with 145 mM NaCl solution and then quickly transferred to a 10 cm petri dish. For fibers that only contain collagen as the core ECM protein, the fibers were briefly washed in general growth media for DFAT cells, immersed in fresh general growth media, and incubated in 37°C incubator for 15 minutes to allow collagen to solidify. For all other fibers that contain fibrinogen as the core ECM protein, the fibers were immersed and incubated with 4.2 units/ml thrombin (T7513-500UN, Sigma-Aldrich Japan, Tokyo, Japan) dissolved in 20 mM HEPES buffered saline with 2 mM CaCl_2_ for 15 minutes at 37°C to convert fibrinogen into fibrin (and allow collagen to solidify at the same time). After incubation with thrombin, the fibers were then briefly washed in general growth media for DFAT cells. All fibers were finally cultured in control media (See Materials and Methods section “Differentiation Induction”) and maintained in a humidified incubator with 5% CO_2_ at 37°C with media exchanged every three or four days. Unless otherwise specified, all fibers used in the experiments were roughly between 300 μm and 350 μm in diameter.

### Measurement of the Stiffness of ECM Proteins

The seven types of ECM proteins, PCol, ACol-L, ACol-H, Fib-L, Fib-M, Fib-H, and ACol-Fib were gelated for measurement of their mechanical stiffness by atomic force microscopy. For gelation, 2 ml of each of PCol, ACol-L, and ACol-H were prepared in 35 mm dishes and incubated at 37°C for 15 minutes. For conversion of fibrinogen to fibrin, 1.33 ml of each of Fib-L, Fib-M, and Fib-H were prepared in 35 mm dishes, mixed with 0.67 ml of 4.2 units/ml thrombin solution, and incubated at 37°C for 15 minutes. ACol-Fib was prepared as a mixture of 1 ml of ACol-H solution containing 100 mM sodium gluconate and 0.67 ml of the stock 33.3 mg/ml fibrinogen solution mixed with 0.33 ml of the 4.2 units/ml thrombin solution in a 35 mm dish and incubated at 37°C for 15 minutes. After gelation, all seven samples of these ECM proteins were exposed to 1 ml of the general growth media added to each of the 35 mm dishes and kept in a humidified incubator with 5% CO_2_ at 37°C overnight for media to diffuse into the gelated ECM proteins and reach equilibrium before the measurements. The stiffness of each sample of these ECM proteins was measured in media with an atomic force microscope (AFM, NanoWizard, JPK Instruments, Berlin, Germany) on top of an inverted microscope (IX71, Olympus, Tokyo, Japan). The AFM cantilever tip was constructed by gluing a 48 μm glass bead (VitraBio, Steinach, Germany) to the end of a silicon cantilever (TL-CONT, NanoSensors, Neuchatel, Switzerland) with a spring constant of 0.3 N/m. The cantilever tip was moved at 3.0 μm/s and pushed to the surface of the gelated ECM protein samples to measure the stiffness. For each sample, the measurement was conducted at 25 points (5 × 5 grid with 25 μm gaps) and repeated for 2 times at each point. The data were analyzed by a built-in software (JPK Data Processing, JPK Instruments, Berlin, Germany). Young’s moduli were obtained by fitting the Hertz model to the force curve.

### Differentiation Induction

Two days following fiber fabrication, DFAT cell fibers were separated into the control group and the differentiation induction group. Control media is basically general growth media (DMEM + 10% HI-FBS + 1% P/S) supplemented with 20 μg/ml of aprotinin (fibrinolysis inhibitor [18]). Smooth muscle differentiation induction media is composed of DMEM + 5% HI-FBS + 1% P/S + 5ng/ml TGFβ supplemented with 20 μg/ml of aprotinin. Media exchanges were performed every three or four days.

### Viability Assay for DFAT Cells in Cell Fibers

Viability assay was performed on DFAT cells in cell fibers 7 and 21 days after differentiation induction using LIVE/DEAD Viability/Cytotoxicity kit (Life Technologies, Carlsbad, CA, USA). DFAT cell fibers from both the control and differentiation induction groups were incubated in live/dead viability assay working solution (1 μM calcein AM + 1 μM ethidium homodimer-1 in PBS (+)) for 30 minutes at 37°C. DFAT cell fibers were subsequently imaged by phase contrast and fluorescence microscopy (IX71, Olympus, Tokyo, Japan).

### Immunocytochemistry

7 and 21 days after differentiation induction, DFAT cell fibers were fixed in 4% paraformaldehyde for 20 minutes at room temperature, during which time the alginate shell dissolved, leaving behind only the core DFAT cell fibers that were subjected to immunostaining. After fixation, DFAT cell fibers were washed in tris-buffered saline (TBS) 3 times, permeabilized with 0.2% Triton X-100 for 5 minutes, and blocked with 5% skim milk for 20 minutes in room temperature. DFAT cell fibers were then incubated with monoclonal mouse anti-ASMA antibodies (1:300 dilution, M085129, Dako Japan, Tokyo, Japan) or monoclonal mouse anti-calponin antibodies (1:300 dilution, C2687, Sigma-Aldrich Japan, Tokyo, Japan) at 4°C overnight with gentle rocking, followed by incubation with Alexa Fluor 568 goat anti-mouse IgG (H+L) antibodies (1:500 dilution, A-11004, Molecular Probes, Life Technologies) for 1 hour at room temperature. Finally, DFAT cell fibers were washed in TBS 4 times and stained with 5 μg/ml of Hoechst 33342 (Sigma-Aldrich Japan, Tokyo, Japan) for 30 minutes at room temperature. Stained cell fibers were imaged by confocal microscopy (LSM 780, Zeiss, Oberkochen, Germany). The percentages of ASMA-positive and calponin-positive cells were estimated by manually counting individual cells from confocal section images of the immunostained cell fiber samples. The total number of cells counted for each condition is at least 95 cells.

## Results and Discussion

### Formation of DFAT Cell Fiber

**Fig 2:**
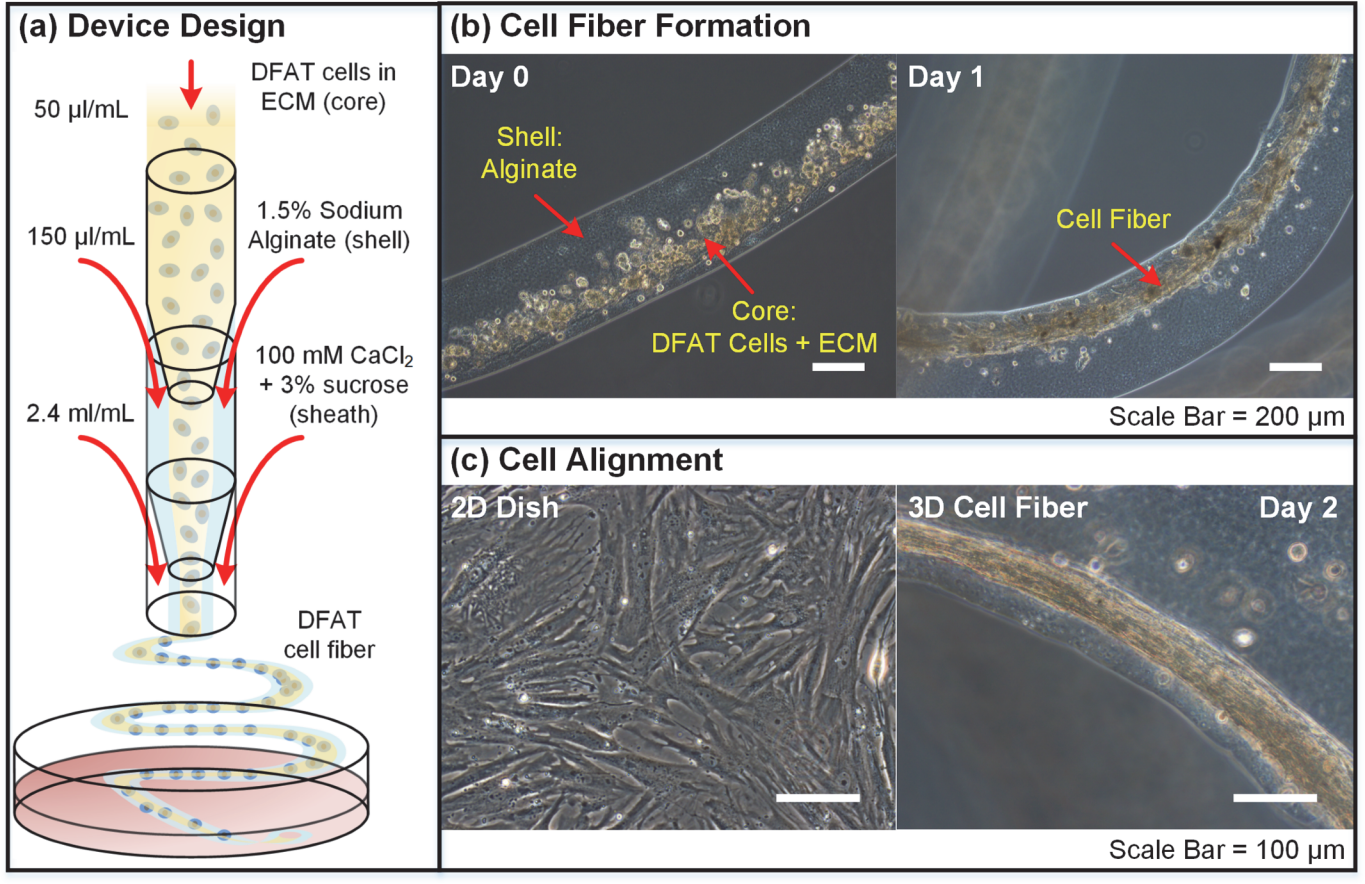
Formation of DFAT cell fiber. (a) Schematic drawing of the microfluidic double co-axial device used to encapsulate cells into the core of alginate fibers with the default flow rates. The inner core stream consist of DFAT cells suspended in the appropriate ECM proteins. The outer shell stream is sodium alginate solution that solidifies when it comes in contact with the sheath stream of calcium chloride solution. (b) Cell fiber formation overnight. A day 0 image captured immediately following fiber fabrication with single DFAT cells dispersed in ECM proteins encapsulated in the center core of the alginate fiber, and a day 1 image showing a section of the formed DFAT cell fiber inside alginate shell. (c) Images comparing DFAT cell alignment when cultured in conventional 2D dish and as 3D fiber (day 2) structure. DFAT cells are randomly oriented when attached on 2D dishes, whereas DFAT cells are highly aligned along the longitudinal fiber direction when cultured as 3D fiber structure.

DFAT cell fibers were formed by the cell fiber technology using the hydrogel core-shell microfiber fabrication device (Fig. 2A). This microfluidic device generated two co-axial laminar flow streams consisting of a core stream of DFAT cells suspended in ECM protein and a shell stream of sodium alginate that solidified upon contacting the sheath stream of CaCl_2_. As a result, DFAT cells suspended in ECM protein were successfully encapsulated inside the core region of calcium alginate shell fibers (Fig. 2B). Within 1 day of culture, DFAT cells stretched and formed into fiber constructs in the core region provided that a stiff enough ECM protein was used (Fig. 2B). The DFAT cell fibers inside the alginate shell were approximately 80 ± 25 μm (mean ± standard deviation) in diameter and 0.5 meter in length. Compared to the random orientation of cells cultured on conventional 2D dishes, DFAT cells cultured as 3D cell fibers were highly aligned in the longitudinal direction (Fig. 2C). These results indicate that the cell fiber technology is a useful method to encapsulate adherent cells in a long and narrow micro-tubular space to restrain cells’ orientation for precise control of cellular alignment in 3D culture. The mechanism underlying such alignment can very likely be attributed to the alignment of collagen and fibrin fibers during flow. It has been shown that collagen fibers orient to the direction of flow when flowed through a microfluidic channel (< 100 μm) [19,20] and fibrin fibers’ alignment to the direction of flow increases with increased flow rate [21]. Since cells tend to spread along the direction of collagen fibers [19], these aligned core ECM proteins in turn serve as the substrates that aid cellular alignment within the microfibers’ micro-tubular space. The cell fiber technology’s advantage of being able to control the cellular orientation at the microscale level allows for more accurately structured hierarchical assembly into larger complex tissues that rely on well-defined intricate cellular orientation for proper functioning.

### Optimization of ECM Protein to Form DFAT Cell Fiber

**Fig 3:**
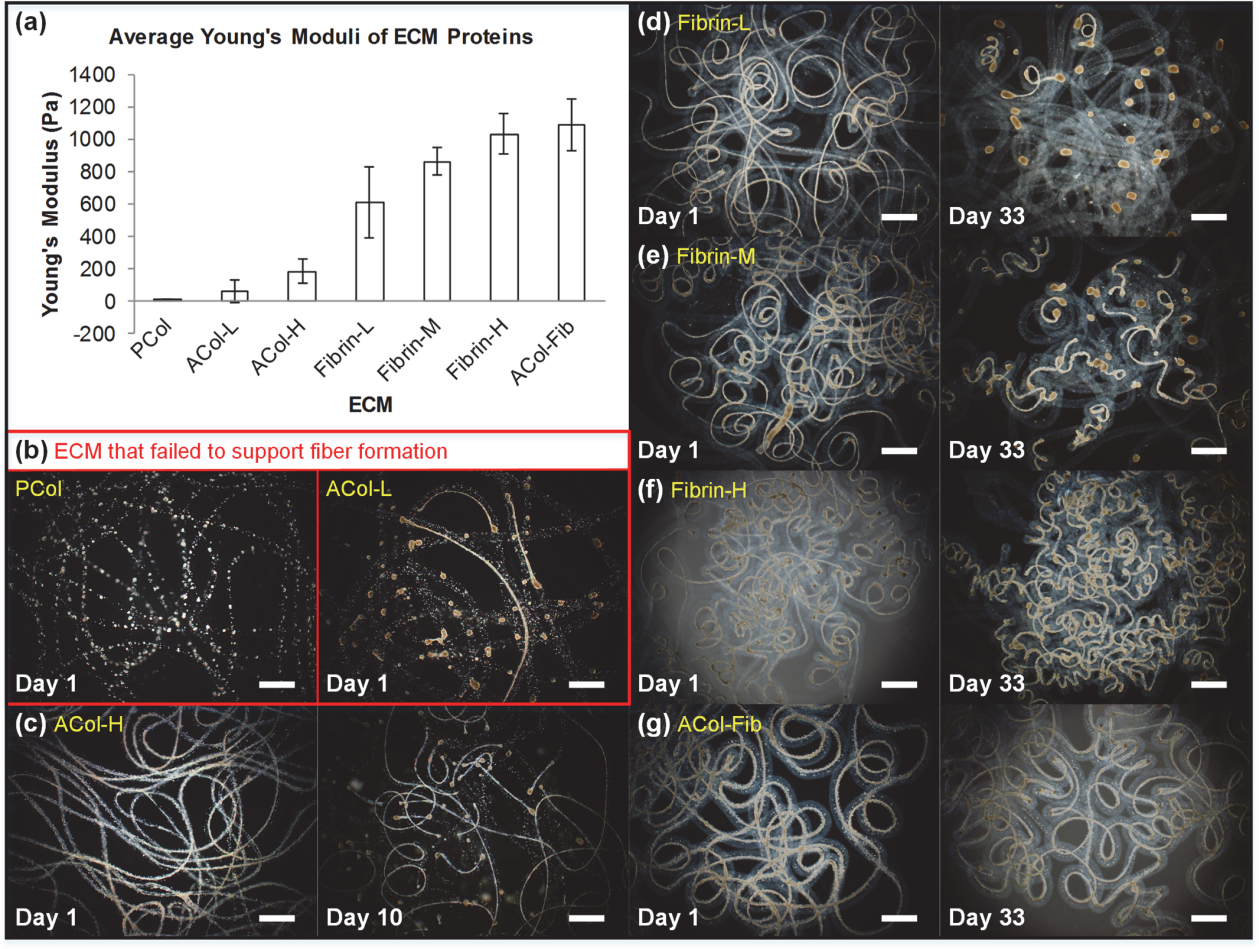
Core ECM protein optimization. (a) Bar graph of the average Young’s modulus of various types of ECM protein, organized in increasing stiffness. n = 50, and data are expressed as the mean ± standard deviation. (b) PCol and ACol-L failed to support fiber formation as shown in the day 1 images where DFAT cells mainly aggregated into spheroids as opposed to cell fibers. (c) ACol-H is stiff enough to support initial formation of DFAT cells into fiber structures, but failed to maintain the fiber structures as almost half of the cells already aggregated into spheroids by day 10. All concentrations of fibrin tested successfully supported initial DFAT cell fiber formation (day 1), but by day 33, almost all cells in Fibrin-L (d) and about half of the cells in Fibrin-M (e) aggregated to spheroids, and only Fibrin-H (f) still maintained the fiber structures. (g) Mixture of collagen and fibrin, ACol-Fib, is also able to support initial DFAT cell fiber formation as well as maintain the fiber structures for at least 33 days. Scale bar = 1 mm.

To investigate the influence of the stiffness of ECM proteins on DFAT cell fiber formation, seven different types of ECM proteins that possess various Young’s moduli were prepared (Fig. 3A). PCol is the least stiff, followed by ACol at increasing concentrations (ACol-L, ACol-H), and then fibrin at increasing concentrations (Fibrin-L, Fibrin-M, Fibrin-H). The mixture of collagen and fibrin (ACol-Fib) sample has the highest Young’s modulus of about 1,000 Pa. DFAT cells suspended in all seven types of ECM proteins were encapsulated in the core region of alginate fibers and cultured for at least 30 days or until the cell fibers shrank into spheroids. When the least stiff PCol and ACol-L were used as the core ECM protein, DFAT cells failed to form cell fiber constructs and mainly formed into spheroids instead (Fig. 3B). When the concentration of ACol doubled to 4 mg/ml (ACol-H) with the Young’s modulus tripled, DFAT cells were able to stretch within this stiffer ECM proteins and form into long continuous cell fibers 1 day after encapsulation (Fig. 3C). However, by day 10 more than half of the cell fiber sections shrank into spheroids. Compared to ACol-H’s Young’s modulus of 180 Pa, the Young’s moduli of the 3 types of fibrin ranged from 600 Pa to 1000 Pa. Although all 3 types of fibrin supported initial formation of DFAT cell fibers (Fig. 3D-F), only Fibrin-H was able to maintain DFAT cell fibers as fiber constructs for over 30 days without shrinkage into spheroids (Fig. 3F). The ECM protein mixture of collagen and fibrin, ACol-Fib, that has a similar Young’s modulus as Fibrin-H also yielded similar results in terms of fiber formation and maintenance over time (Fig. 3G). These results indicate that the success of DFAT cell fiber formation is dependent on the mechanical stiffness of the supporting ECM proteins reaching a threshold of 180 Pa and that DFAT cell fiber maintenance for more than 30 days can be achieved by the use of ECM proteins with a mechanical stiffness of around 1000 Pa. We have shown that cell fiber formation varies when different core ECM proteins are used. Since different cells possess different mechanical and biological properties and respond differently to substrates with varying rigidity [22], the choice of a proper core ECM protein is a key to form cell fibers consistently. In this paper, we successfully found that mechanical stiffness is one of the properties of core ECM proteins that affects the success of cell fiber formation. This finding could be supported by a previous report showing that substrate stiffness influences the adhesion and spreading of cells [16]. When the stiffness of the ECM protein does not reach a certain threshold, DFAT cells might not be able to stretch within the ECM protein nor anchor to a stiff substrate; as a result, they tend to aggregate with themselves and form into spheroids. For practical application, we selected the collagen and fibrin mixture, ACol-Fib, over Fibrin-H as the core ECM proteins for consistent DFAT cell fiber formation because the collagen in ACol-Fib provides the higher viscosity that can prevent DFAT cells from settling down and causing non-uniformity in cell density of the core solution during core-shell microfiber fabrication.

### Self-Assembly of DFAT Cell Fibers into Coiled Spring Structures

**Fig 4:**
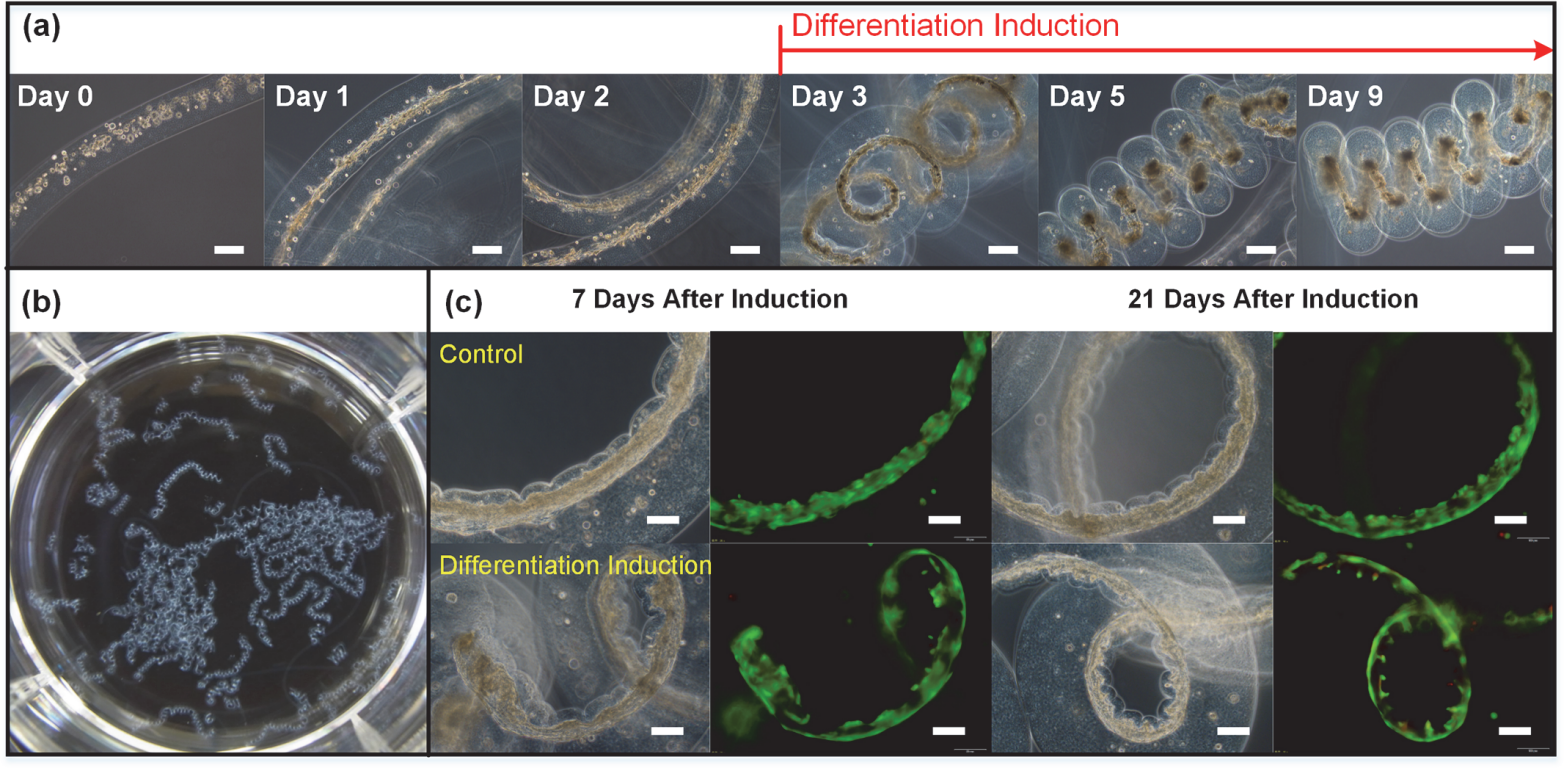
Self-assembly of DFAT cell fibers into coiled spring structures. (a) Images showing DFAT cell fiber formation in the first 2 days and self-assembly into coiled spring structures upon differentiation induction on day 2 of culture. Scale bar = 200 μm. (b) An actual image of the fibers in a 6-well plate, showing the uniformity of the coiled spring shapes. (c) Viability assay performed on DFAT cell fibers 7 and 21 days after differentiation induction. Phase and fluorescent images of DFAT cell fibers show that most cells are alive (green) with minimal amount of dead (red) cells. Scale bar = 100 μm.

To investigate whether DFAT cell fibers self-assemble into spring structures during the differentiation process, DFAT cells in the cell fibers were induced to differentiate to the smooth muscle cell lineage in differentiation induction media. DFAT cells suspended in ACol-Fib and encapsulated in alginate shell were allowed to form into the cell fiber construct for 2 days. After the media was changed to differentiation induction media, DFAT cell fiber started to coil on Day 3 (Fig. 4A). By day 5, the DFAT cell fiber has further coiled into a uniform spring structure that is kept through the end of culture period. During this time, proliferation of DFAT cells and their differentiation into smooth muscle cell lineage could contribute to an increase in the overall cell traction force exerted by the DFAT cell fiber. Since in most cases DFAT cell fibers preferentially form and position to an off-center side of the core-shell microfibers, the increase in the cell traction force of DFAT cell fibers concentrated on one side of the core-shell microfibers might cause the microfibers to bend and eventually led to the coiled spring structures. Next, we observed fiber sections in a dish to determine the consistency and uniformity of the self-assembled cell spring structures. Fig. 4B shows an actual image of various DFAT cell fibers self-assembled into spring structures in a 6-well plate, highlighting the consistent formation and uniformity of the spring structures across long fiber sections and among separate fibers. Because the construction of the spring structures from DFAT cell fibers is achieved through a self-assembly process, it is important to control the uniformity of the linear cell fibers before differentiation induction. When cells were evenly suspended in ECM proteins and distributed throughout the lengths of the fibers, they naturally form into cell fiber constructs with uniform thickness along the entire lengths of the fibers. Such attribute led to the generation of similar amount of contractile force by DFAT cells along the entire fiber length, and therefore gave rise to the self-assembly of very uniformly shaped and coiled cell springs. The key to make uniformly coiled spring structures greatly depends on the uniformity of the initial cell encapsulation. We have observed the formation of perfectly coiled spring structures having total lengths of up to approximately 2800 μm. Finally, 7 and 21 days after differentiation induction, viability of the control and differentiation induced cells in the fiber constructs was evaluated. Most of the cells in the DFAT cell fibers cultured in both control and differentiation induction media were still alive 7 and 21 days after differentiation induction as shown in Fig. 4C.

### Characterization of Coiled Spring Formation and Structure

**Fig 5:**
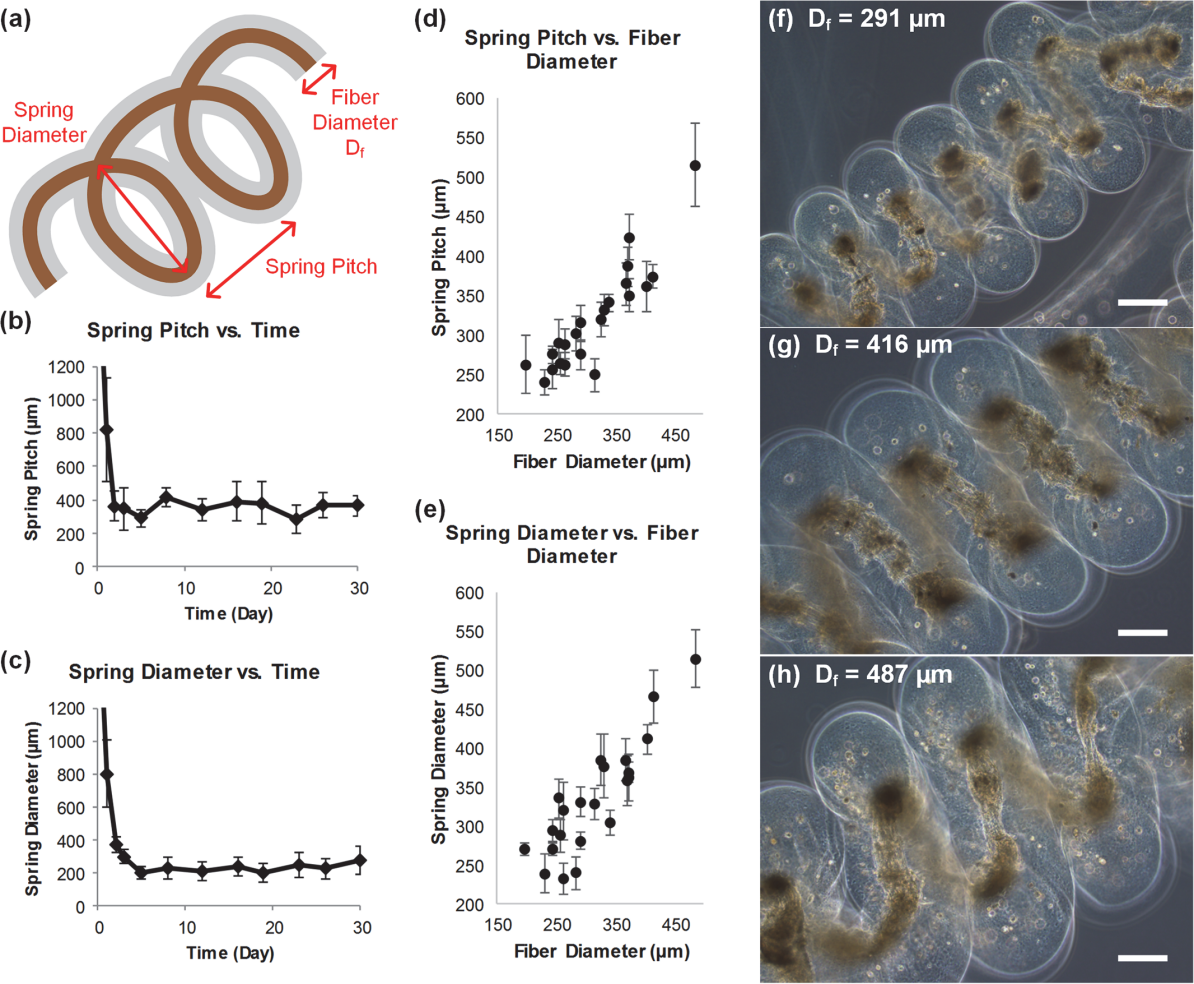
Cell spring characterization. (a) Schematic image defining various parameters of a spring. (b) Graph showing the spring pitch over 30 days of culture. The spring pitch reached about 300 μm by day 2 or 3 of culture and remains constant. n = 9, and data are expressed as the mean ± standard deviation. (c) Graph showing the spring diameter over 30 days of culture. The spring diameter decreased to about 200 μm by day 5 and stays constant for the rest of the culture period. n = 10, and data are expressed as the mean ± standard deviation. (d) Spring pitch and (e) spring diameter as functions of fiber diameter D_f_. As the fiber diameter increases, both spring pitch and diameter also increase. n ≥ 3, and data are expressed as the mean ± standard deviation. (f), (g), (h) Images of cell springs with D_f_ = 300, 400, and 500 μm, respectively. Scale bar = 200 μm.

To examine whether the cell spring structures change over time, we measured the spring pitch and spring diameter for a 30-day period (Fig. 5A). Regarding the spring pitch, after 2 or 3 days of culture as the cell traction forces increased, DFAT cell fibers already self-assembled into the most compact form where there was no more space between the successive coils (Fig. 5B). This means that the space between the coils (or spring pitch) roughly equaled to and was therefore limited by the fiber diameter. Since the spring pitch is limited by the fiber diameter, it remained constant for the rest of the culture period. Next, the change in the spring diameter over time was analyzed (Fig. 5C). As a linear fiber slowly formed into a spring shaped construct in the first 3 days, the diameter decreased as expected. After the spring pitch reached its minimum by day 3, there was already no more space between the successive spring coils. When the cells’ inherent traction forces continued to increase as the cells slowly differentiated into the smooth muscle cell lineage, the spring diameter continued to decrease until day 5 when the diameter also plateaued and stayed constant for the rest of the culture period.

We also characterized the relationship between the original fiber diameter and the spring shape (pitch and diameter) to explore whether the spring shape can be controlled by the fiber diameter. Fig. 5D-E indicate that both the spring pitch and spring diameter increased when the original fiber diameter increased. This relationship can also be seen from the actual DFAT cell spring images shown in Fig. 5F-H. Since both the spring pitch and spring diameter positively correlate with the fiber diameter, the spring shape can be controlled by changing the original fiber diameter at the fiber fabrication step (which is determined by the device design and flow rate parameters). By scaling up the size of the fiber fabrication device, fibers of up to 1000 μm in diameter can be achieved. As the final application of these cell springs is to serve as tissue engineering building blocks to reconstruct circumferential smooth muscles of various organs, control over the design of the spring size and shape makes the system more versatile.

### Expression of Smooth Muscle Specific Markers

**Fig 6:**
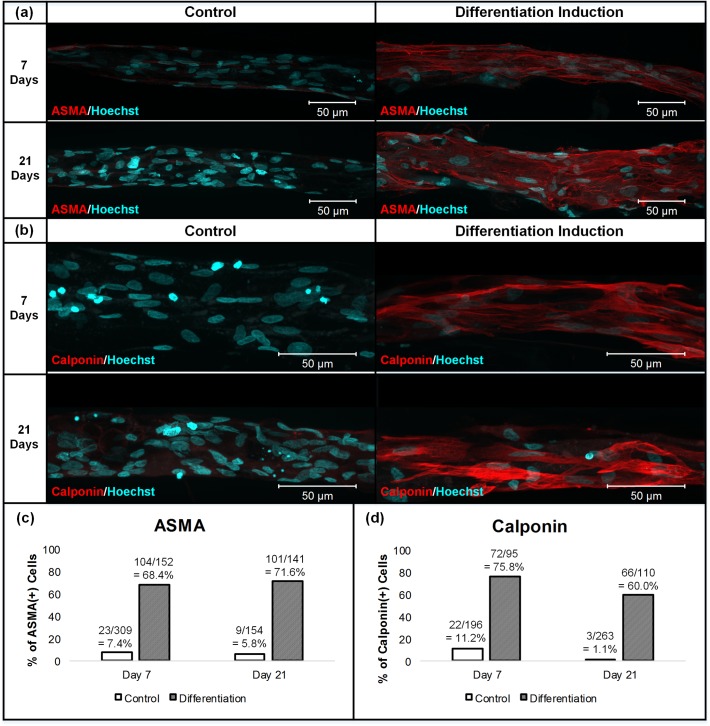
Expression of smooth muscle specific markers. Maximum intensity projection images of control and differentiation induced DFAT cell fibers immunostained with ASMA (a) or calponin (b) taken by confocal microscope. Bar graphs of the percentages of ASMA-positive (c) and calponin-positive (d) cells as estimated from confocal images. The total number of cells counted from confocal section images of immunostained cell fibers for each condition is at least 95 cells. Overall, DFAT cell fibers cultured in differentiation induction media express higher percentages of ASMA-positive and calponin-positive cells than when cultured in control media.

To confirm the differentiation of DFAT cells to the smooth muscle lineage, DFAT cell fibers were evaluated for their expression of smooth muscle specific markers by immunostaining 7 and 21 days after differentiation induction. Fig. 6A shows the maximum intensity projection images of DFAT cell fibers immunostained with anti-ASMA (alpha smooth muscle actin, an early smooth muscle lineage specific marker [1,14,23]) antibodies. Compared to the DFAT fibers cultured in control media, those cultured in differentiation induction media clearly contained higher percentages of ASMA-positive cells with well-aligned ASMA expressed uniformly throughout the fiber sections. The high percentages of ASMA-positive cells in the DFAT cell fibers cultured in differentiation induction media indicated that these cells were differentiating to the smooth muscle lineage. The uniform alignment of ASMA in the longitudinal direction further confirmed that DFAT cells were highly aligned in a single direction when cultured as fiber constructs. Next, the expression of calponin, a mid to relatively late marker of smooth muscle differentiation highly restricted to differentiated smooth muscles [1,24–26], was evaluated. The maximum intensity projection images of DFAT cell fibers immunostained with anti-calponin antibodies are shown in Fig. 6B. Similar to the ASMA results, DFAT cell fibers cultured in differentiation induction media contained much higher percentages of calponin-positive cells compared to those cultured in control media. The calponin expression patterns also reflected the longitudinally aligned orientation of DFAT cells in the fiber constructs. These results further confirmed that these DFAT cells were differentiating to the smooth muscle lineage. The estimated percentages of ASMA-positive and calponin-positive cells are presented as bar graphs in Fig. 6C-D. Approximately 70% of DFAT cells induced to differentiate were found to be ASMA-positive cells, and approximately 60–75% of DFAT cells induced to differentiate were found to be calponin-positive cells.

Previous studies of the differentiation of human and rat DFAT cells to the smooth muscle lineage on conventional 2D dishes also demonstrated the expression of ASMA [14,23] and calponin [23] in DFAT cells after differentiation induction. However, since the DFAT cells cultured on 2D dishes were randomly oriented, the orientations of the ASMA fibers and calponin were also random. Nevertheless, these DFAT cells were further demonstrated to promote smooth muscle regeneration in mouse and rat models [14,23], highlighting the potential of DFAT cells to differentiate into functional smooth muscle cells. Since the cells in our cell fibers not only expressed the same ASMA and calponin markers but also with organized alignment, it is promising that these smooth muscle-like cell fibers would also be able to recapitulate smooth muscle function in *in vivo* models. As the direction of smooth muscle contraction is dictated by the cellular orientation, the precisely aligned smooth muscle-like cells in our differentiated cell fibers have the potential of generating controlled contraction in the direction of interest. The results altogether suggest that these circumferentially oriented smooth muscle-like tissue constructs are promising building blocks for tissue engineering or as therapeutic modalities in regenerative medicine.

### Summary

In this study, we succeeded in forming smooth muscle-like tissue constructs with cells precisely oriented three-dimensionally in the circumferential pattern by differentiating multipotent DFAT cells in hydrogel core-shell microfibers. This achievement is attributed to the successful formation and maintenance of DFAT cell fibers in a fiber-shaped 3D microenvironment containing optimized supporting ECM proteins. By suspending DFAT cells in a relatively stiff matrix inside a narrow tubular space, DFAT cells could align longitudinally and exert uniform contractile forces in a single direction that eventually led to the self-assembly of the cell fibers into coiled spring constructs upon smooth muscle differentiation induction. This method is expected to be applicable to the differentiation of other multipotent cells such as mesenchymal stem cells, embryonic stem cells, and induced pluripotent stem cells to the smooth muscle lineage. Moreover, the self-assembled smooth muscle cell-like spring constructs could be combined with other tissue engineering building blocks such as linear cell fibers and spheroids to reconstruct larger complex tissues.

Our method further offers the efficiency of accomplishing smooth muscle differentiation and circumferential orientation of the cells at the same time through a self-assembly process. By taking advantage of DFAT cells’ increase in cell traction forces upon differentiation induction, coiled spring constructs formed spontaneously without any external manipulation. This self-assembly process avoids any chemical and mechanical stresses that can be caused by the manual handling and assembling processes. Furthermore, manual coiling of linear cell fibers into spring structures at the microscale would often require skilled hands such as those of experienced surgeons. In such case, the final cell spring construct would most likely require extra scaffold (material) [5] to maintain the spring structure without collapsing back to the linear form. Whereas here, our cell springs could be kept through long term culture as ready-to-use tissue engineering building blocks.

Given that DFAT cells have previously been shown to contribute to injured bladder tissue regeneration and improve urethral sphincter contractility in mouse and rat models [14,23], respectively, it is promising that our DFAT cell fibers could also be used to regenerate smooth muscles *in vivo*. With the cell fibers’ high handleability [12], DFAT cell fibers have the potential to be used clinically to treat smooth muscle related disease such as urinary incontinence. Compared to injection of dispersed single cells, the cell fiber constructs allow for more control over the site of implantation, and the already aligned DFAT cells in the fiber constructs are expected to contribute to regeneration of smooth muscle function faster.

## Conclusion

The present study demonstrates a useful method to engineer circumferentially oriented smooth muscle-like tissue constructs. The ability to encapsulate DFAT cells suspended in an ECM protein with appropriate stiffness inside a long tubular space is the key for DFAT cells to align longitudinally and be kept as cell fiber constructs over time. The self-assembly process allows us to form circumferentially oriented cellular structure without using manual assembling that may cause undesired chemical and mechanical stresses to the cells. Being able to control the initial fiber diameter provides additional control over the design of the subsequent self-assembled spring pitch and diameter. This system is anticipated to be applicable to *in vitro* tissue engineering of the wall of blood vessels as well as *in vivo* regeneration of urethral sphincter muscles as treatment of stress urinary incontinence. These characteristics altogether greatly expand the versatility of the system, making the cell spring constructs more adaptable to a wide array of tissue engineering and clinical applications.

## Supporting Information

S1 Fig Orientation of immunostained ASMA and calponin in differentiated DFAT cell fibers. The orientations of ASMA and calponin in differentiated DFAT cell fibers were quantified by the fast Fourier transform (FFT) method [27]. Two square regions from each of the Day 7 and Day 21 differentiated DFAT cell fibers immunostained for ASMA or calponin (Fig. 6) were characterized to generate FFT output images. The gray scale pixels distributed in circular patterns in the FFT output images reflect the ASMA or calponin fiber orientations. The sum of the pixel intensities from each angle show the directional alignment of the analyzed ASMA and calponin fibers. The brightness distribution of the FFT images are plotted in S1 Fig. Both ASMA and calponin fibers were mainly aligned to the 90° angle. (a) Image of immunostained ASMA in DFAT cell fiber induced to differentiate for 7 days with the angle θ defined. (b) and (c) Plots of the brightness distribution of the FFT images analyzed from the Day 7 differentiated DFAT cell fiber immunostained for ASMA. (d) and (e) Plots of the brightness distribution of the FFT images analyzed from the Day 21 differentiated DFAT cell fiber immunostained for ASMA. (f) and (g) Plots of the brightness distribution of the FFT images analyzed from the Day 7 differentiated DFAT cell fiber immunostained for calponin. (h) and (i) Plots of the brightness distribution of the FFT images analyzed from the Day 21 differentiated DFAT cell fiber immunostained for calponin.
(TIF)

S2 Fig Relationships between different fiber parameters. (a) A drawing illustrating the definition of various diameters of the core-shell hydrogel fiber. (b) Core diameter (D_core_) vs. total fiber diameter (D_f_). As expected, as the total fiber diameter (D_f_) increases, the core diameter (D_core_) also increases. (b) Cell fiber diameter (D_cell_) vs. core diameter (D_core_). In general, the cell fiber diameter (D_cell_) roughly correlates with the core diameter (D_core_). As the core diameter (D_core_) increase, the diameter of the cell fiber (D_cell_) that is formed also tend to increase. (c) Cell fiber diameter (D_cell_) vs. total fiber diameter (D_f_). Cell fiber diameter (D_cell_) also positively correlates with the total fiber diameter (D_f_).
(TIF)

S3 Fig Relationships between the core and cell fiber diameters and the cell spring shape (pitch and diameter). (a) Spring pitch vs. core diameter (D_core_) and spring diameter vs. core diameter (D_core_). Both spring pitch and spring diameter have moderately weak positive correlations with the core diameter (D_core_). As the core diameter (D_core_) increases, the spring pitch and the spring diameter also tend to increase. (b) Spring pitch vs. cell fiber diameter (D_cell_) and spring diameter vs. cell fiber diameter (D_cell_). Moderate positive correlations are seen between the spring pitch (and diameter) and the cell fiber diameter (D_cell_).
(TIF)
