# Supplementary figures and images for: Smooth Muscle-Like Tissue Constructs with Circumferentially Oriented Cells Formed by the Cell Fiber Technology
Source: PLoS One. 2015 Mar 3;10(3):e0119010. doi: 10.1371/journal.pone.0119010 (PMC4348165; doi:10.1371/journal.pone.0119010)

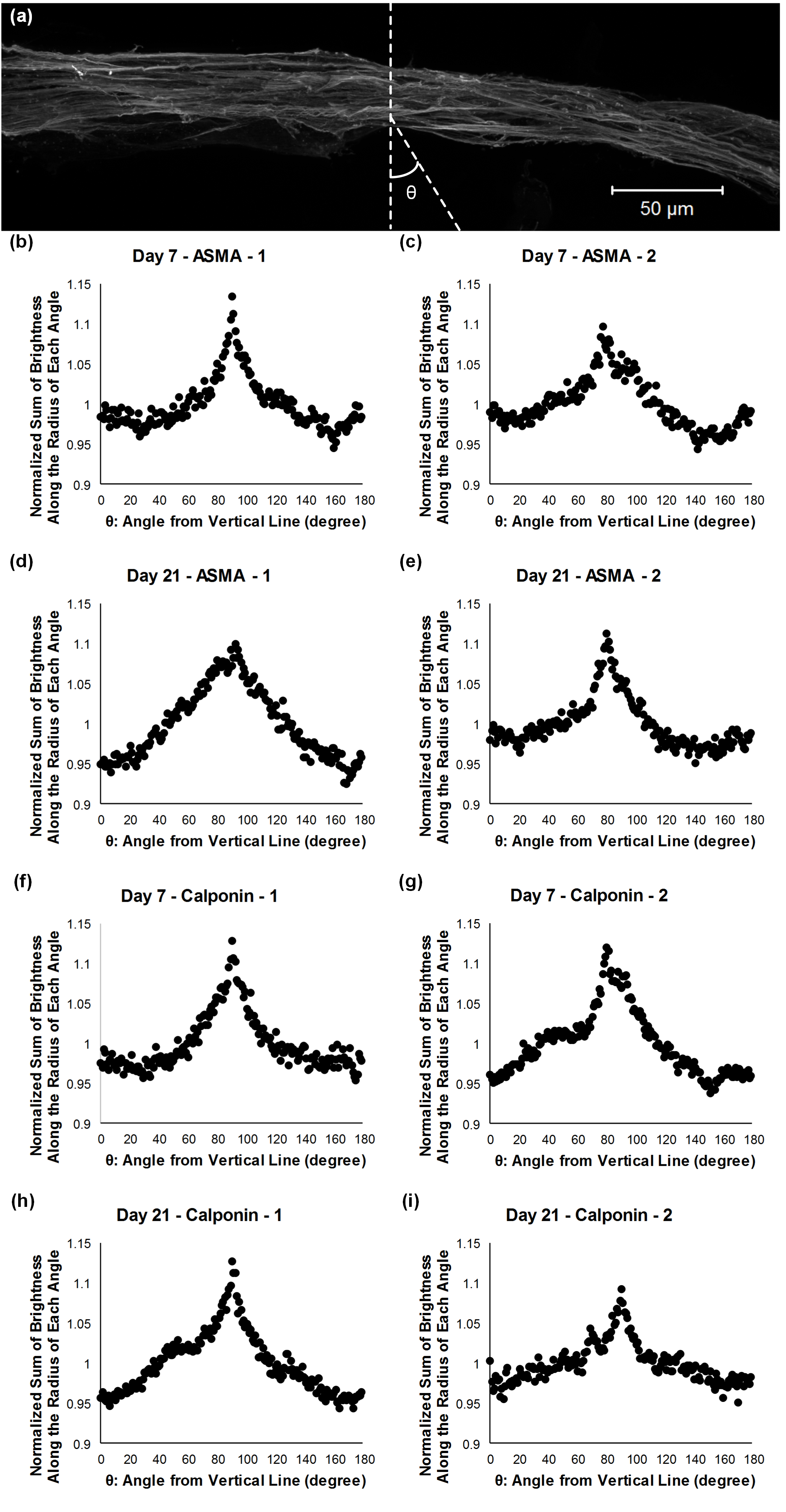

Supplement: S1 Fig — The orientations of ASMA and calponin in differentiated DFAT cell fibers were quantified by the fast Fourier transform (FFT) method [27]. Two square regions from each of the Day 7 and Day 21 differentiated DFAT cell fibers immunostained for ASMA or calponin (Fig. 6) were characterized to generate FFT output images. The gray scale pixels distributed in circular patterns in the FFT output images reflect the ASMA or calponin fiber orientations. The sum of the pixel intensities from each angle show the directional alignment of the analyzed ASMA and calponin fibers. The brightness distribution of the FFT images are plotted in S1 Fig. Both ASMA and calponin fibers were mainly aligned to the 90° angle. (a) Image of immunostained ASMA in DFAT cell fiber induced to differentiate for 7 days with the angle θ defined. (b) and (c) Plots of the brightness distribution of the FFT images analyzed from the Day 7 differentiated DFAT cell fiber immunostained for ASMA. (d) and (e) Plots of the brightness distribution of the FFT images analyzed from the Day 21 differentiated DFAT cell fiber immunostained for ASMA. (f) and (g) Plots of the brightness distribution of the FFT images analyzed from the Day 7 differentiated DFAT cell fiber immunostained for calponin. (h) and (i) Plots of the brightness distribution of the FFT images analyzed from the Day 21 differentiated DFAT cell fiber immunostained for calponin. (TIF) [file pone.0119010.s001.tif]

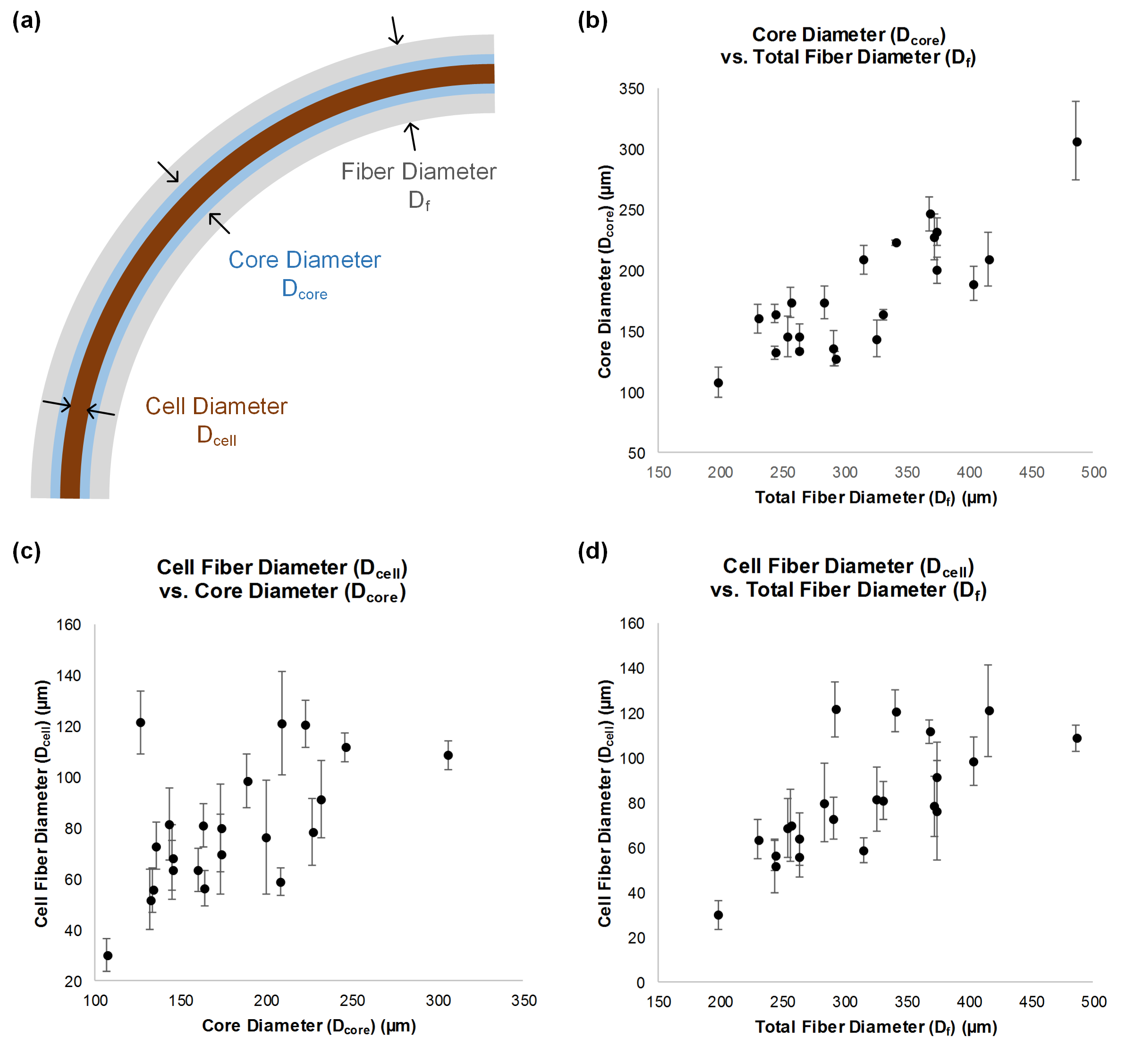

Supplement: S2 Fig — (a) A drawing illustrating the definition of various diameters of the core-shell hydrogel fiber. (b) Core diameter (Dcore) vs. total fiber diameter (Df). As expected, as the total fiber diameter (Df) increases, the core diameter (Dcore) also increases. (b) Cell fiber diameter (Dcell) vs. core diameter (Dcore). In general, the cell fiber diameter (Dcell) roughly correlates with the core diameter (Dcore). As the core diameter (Dcore) increase, the diameter of the cell fiber (Dcell) that is formed also tend to increase. (c) Cell fiber diameter (Dcell) vs. total fiber diameter (Df). Cell fiber diameter (Dcell) also positively correlates with the total fiber diameter (Df). (TIF) [file pone.0119010.s002.tif]

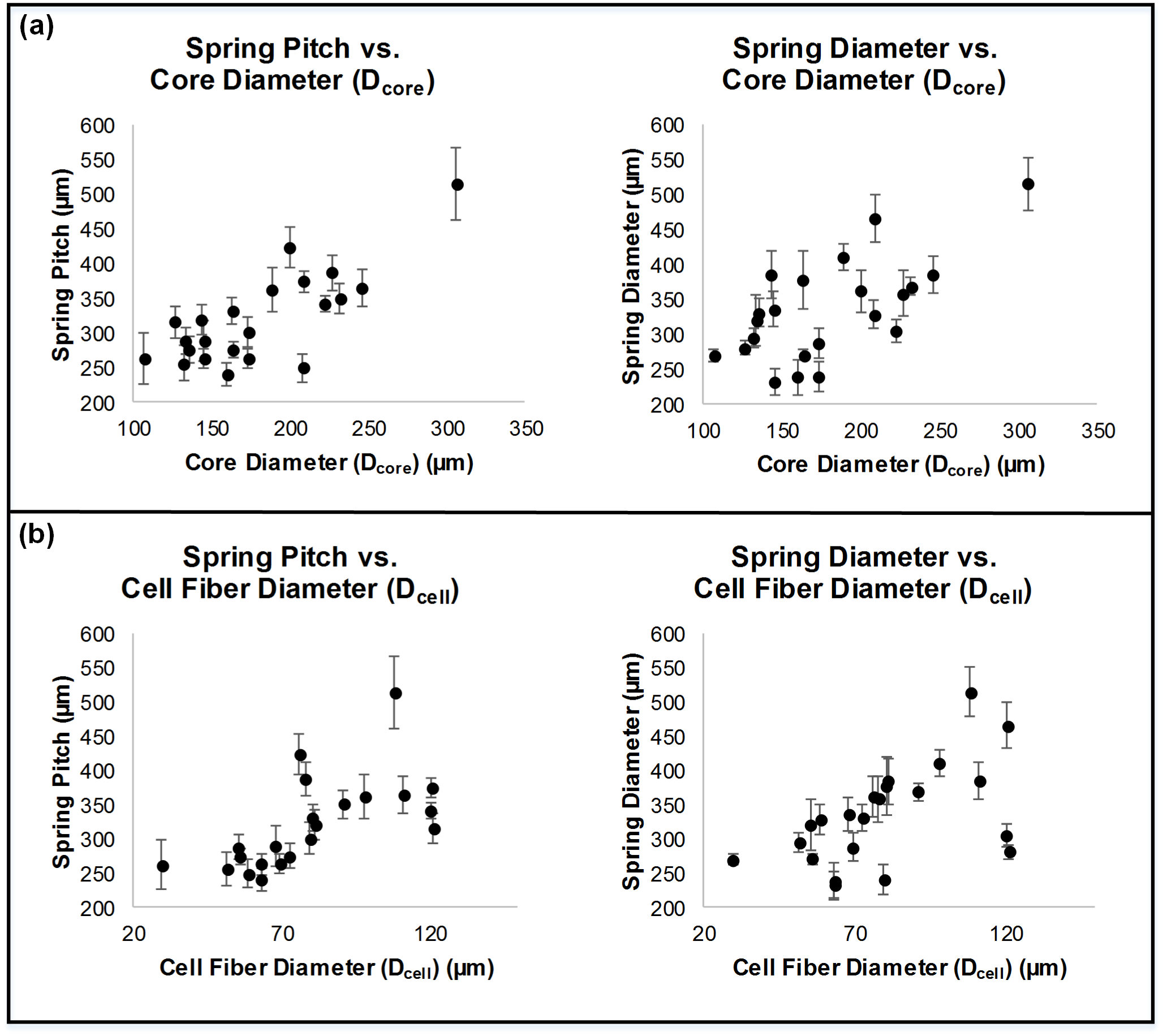

Supplement: S3 Fig — (a) Spring pitch vs. core diameter (Dcore) and spring diameter vs. core diameter (Dcore). Both spring pitch and spring diameter have moderately weak positive correlations with the core diameter (Dcore). As the core diameter (Dcore) increases, the spring pitch and the spring diameter also tend to increase. (b) Spring pitch vs. cell fiber diameter (Dcell) and spring diameter vs. cell fiber diameter (Dcell). Moderate positive correlations are seen between the spring pitch (and diameter) and the cell fiber diameter (Dcell). (TIF) [file pone.0119010.s003.tif]
